# Needs assessment of emergency medical and rescue services in Abuja/Nigeria and environs

**DOI:** 10.1186/s12873-019-0291-9

**Published:** 2019-12-05

**Authors:** Peter Asaga Mac, Axel Kroeger, Philomena Ehi Airiohuodion

**Affiliations:** 1 Centre for Medicine & Society (Global Health), University Medical Centre Freiburg, 79014 Freiburg, Germany; 2 Institute of Human Virology, University Medical Centre Freiburg, Freiburg, Germany; 3 Department of Paediatrics, Zankli Medical Centre, Abuja, Nigeria

**Keywords:** Emergency medical rescue services (EMRS), Delay times, Abuja, Road traffic injury (RTI), NEMA, FRSC, MAITAMA

## Abstract

**Background:**

Nigeria is ranked second highest in the rate of road accidents and other emergencies (Deaths, disabilities) among 193 countries of the world. There is therefore the need for analyzing Emergency Medical Rescue Services (EMRS) in the country to identify options for improvement.

**Method:**

The study was conducted from February, 2016 to March, 2017 in three EMRS organizations (FRSC, NEMA and MAITAMA Hospital) located in Abuja. The structure, resources, process of EMRS activities and outcome (delay times, case fatality as well as victims and service-providers satisfaction with services) were assessed through observation, time measurements and interviews.

**Results:**

FRSC and NEMA offers (Road Traffic Injury) RTI and Disaster services, the ambulances consist of Intensive Care Unit(ICU) buses, Helicopters, Speed boats, motorbikes and other specialized vehicles. Mortality and morbidity recorded for 2016 was 1.1 and 2% respectively. MAITAMA is a specialist centre that offers general medical services. A total number 1227(88.8%) lives were saved during the observational period by three organizations, 60(4.9%) deaths, 132 (9.6%) disabilities, 793 (57.2%) NCDs and 593(42.8%) RTI.

**Conclusion:**

Non-communicable diseases (NCDs) cause many deaths and morbidities in the developing world compared to infectious diseases. There is need for total revamping and education of EMRS institutions in Nigeria and Low- Middle Income Countries (LMICs). Abuja and its surroundings suffers from delays in rapid emergency services, lack of adequate awareness, functional ambulances, minimal specialists and inadequate consumables lead to the loss of many lives.

## Background

The three fundamental functions of a health system are to improve the health of the population, respond to people’s expectations, and provide financial protection against the costs of ill-health [1, 2]. Emergency medical care can contribute positively to these functions. There are no empirical data on the number of lives or disability-adjusted life-years (DALYs) saved through emergency medical care [2–4]. Nevertheless, many of the conditions that contribute to the burden of disease in Nigeria and other low-income countries can be mitigated through prompt treatment [5–7]. Enhancing a health system’s responsiveness to people’s expectations leads to improved utilization of services and better outcomes [1, 8]. Access to medical care for urgent or life threatening conditions is a key expectation in many communities in Nigeria [9–11]. In response to this, two major institutions were created in addition to the existing emergency departments in the major governmental hospitals. These are:

### Federal Road Safety Commission (FRSC)

The Federal Road Safety Commission (FRSC) was established in 1988. The agency is the lead organization in Nigeria on road safety administration and management and has offices in all States of the country. The statutory function of the agency includes; making the highways safe for motorist and other road users; checking road worthiness of vehicles; recommending works and infrastructures to eliminate or minimize accidents on the highways and educating motorists and members of the public on the importance of road discipline on the highways [12]. It has vehicle ambulances equipped with medical gadgets and devices. FRCS has a call centre with a unique number which is toll free. The services are free of charge.

### National Emergency Management Agency (NEMA), Abuja Nigeria

NEMA was established in the year 1999, and is saddled with the responsibility of managing medical emergencies such as fire outbreaks, disease epidemics, flood disasters and road traffic accidents [13, 14]. It is a federal government agency that is promptly mobilized during disasters and other medium scale emergencies. The agency is equipped with modern emergency combating gadgets such as Helicopter; Vehicle and Boat ambulances. NEMA has a call centre with a national emergency number which is 24 h toll free. The agency also has Beacon services (a tracking transmitters, which is triggered during an emergency). The basic purpose of this system is to help rescuers find survivors within the so-called “golden day”(the first 24 h following a traumatic event) during which the majority of survivors can usually be saved. The ambulance services are free of charge. The agency has offices located in the 36 states of the Federal Republic of Nigeria.

### MAITAMA hospital Abuja

The Hospital is located along Aguyi Ironsi Way, Maitama district, Abuja. It is one out of the many federal government owned tertiary care medical centres with the responsibility of providing general medicine and specialized services. The medical team includes 15 medical consultants in various fields of medicine including accident and emergencies (A&E). It has a well-functioning unit of accident and emergency care, with vehicle ambulances equipped with medical equipment and first aid devices [15]. The accident and emergency unit call centre is called” Compound Unit” which operates a 24-h service. Their emergency number is different from those of the other institutions. The three institutions work synergistically. The purpose of this study is to analyse different aspects of the medical emergency system in Abuja and Keffi in order to identify possible weaknesses and limitations which could be corrected by policy decisions and practical actions in order to make the life safer for the local population.

## Methods

This study was conducted in Abuja federal capital territory and Keffi area council of Nasarawa state. Abuja is the federal capital and the seat of federal government of Nigeria. It has a total population of 3.4 million people and an area of 1769 km^2^ with a population density of 698.6 inhabitants/km^2^ [16].

### Documentation of published and unpublished materials

An analysis of locally available documents and a literature review was done to get information on the burden of medical emergencies dealt with by FRSC, NEMA and MAITAMA hospital. A literature review on relevant information on medical emergencies with a focus on low income countries was done. Publications were identified through search engines; unpublished material included hospital and EMRS booklets, files and other records in the three institutions. Photos were taken during the field visits to the EMRS agencies and rescue operations.

### Qualitative methods

Focus Group discussions (FGDs) with EMRS providers (mainly paramedics, nurses and doctors) and organizers (mainly team managers and administrative staff) were conducted, one FGD for each organization with about 10 participants in each group. Personal interviews (both semi-structured questionnaires and in-depth interviews) were conducted by the Principal Investigator (PI) and trained interviewers with 1500 victims and/or their relatives. Furthermore, in-depth interviews were conducted by the PI with heads of three main agencies offering EMRS (NEMA, FRSC, and MAITAMA Hospital).

### Participant observations, interviews and time measurements (qualitative/ quantitative)

Measurements of delay times were carried out (from receiving *the emergency call to attending to the patient in the emergency ward*) by the principal investigator by accompanying for 2 weeks the emergency team/staff of three institutions, observing their activities while rescuing victims and measuring of the delay times between call and arrival at scene. Also, the activities of the Bay Post staff were observed using an observation list (A Bay post is a road side building that houses emergency ambulances and personnel, located every 17 km along major roads in Abuja; see below). At the Call Centres interviews were conducted and forms were checked to document the number of calls per shift. Participant observations were made during and after the rescue operations regarding people’s awareness of the emergency system. The EMRS Basic equipment in ambulance services were observed and documented.

### Questionnaire survey and data analysis

A questionnaire with closed and open-ended questions was applied to EMRS users and providers to extract information on the quality of EMRS activities as perceived by victims, their relatives and emergency care providers. It included questions on the frequency of EMRS refresher training courses for improving skills in Basic Life Support (BLS), Advanced Cardiac Life Support (ACLS) and Advanced Trauma Life Support (ATLS). Responses to the closed questions were entered into the computer and analysed with SPSS package producing descriptive statistics. Responses to open ended questions were transcribed and analysed using categorical evaluation.

### Ethical considerations/statement

The research study was conducted in compliance with the ethical principles of the Nuremberg Code and the Declaration of Helsinki (BMJ, 1996; WHO, 2001). The research protocol was submitted and approved by the Ethical Committee of the University of Freiburg (approval number 171/17) which was accepted by the Nigerian authorities. Written informed consent was obtained from all study participants.

## Results

Table 1 summarizes the findings of the document analysis of the three EMRS organizations in Abuja/ Keffi. It shows that FRSC is responsible for rescue operations related to traffic accidents, NEMA for natural disasters and MAITAMA for general medical emergencies. The staff numbers are highest in FRSC with 19,000 employees in the whole country most (64.7%) of them being males, second NEMA with 700 employees, predominantly males (64.7%), and third MAITAMA where most of the 762 staff were nurses (72.3%). The emergency transport system of the 3 EMRS organizations were well coordinated, equipped with most of the basic emergency rescue equipment. Rescue services are free and funded by the government.

**Table 1:** Resources and main services of the three institutions In Nigeria (2016). Equipment and staffing of Bay posts, ambulances and boats

INST/ORG	FOUNDING YEAR	SERVICES	AMBULANCE TYPES	NO OF STAFF AND % GENDER DISTRIBUTION		
FRSC	1988	Road safety	ICU Buses	19,000	Female (35.3%)	Male (64.7%)
NEMA	1999	Disaster Mgt.	ICU Buses, Helicopters, Boats, motorbikes.	700	Female (35.3%)	Male (64.7%)
MAITAMA	2001	General health services.	. ICU Buses	762	Female (72.3%)	Male (27.7%)

The information was obtained in the three call centres; observations were made in 6 Bay posts, 32 ambulances and 2 boats.

i) *The Bay-Post system:* Road safety in Abuja and Keffi is largely offered by Bay posts. These are road side buildings located every 17 km along the major roads in Abuja and other cities in Nigeria. Bay posts (see photo) have ambulance buses and motorbikes equipped with Intensive Care units (ICU). The staff of a Bay post includes Paramedics and Nurses (Table 2). Bay posts are operated by NEMA and FRSC but not by MAITAMA. When an emergency call is received by the call centre, the operator will alert the closest Bay post to the scene to deploy ambulances. FRSC and NEMA have a total of 23 Bay Posts located within and on the outskirts of Abuja/Keffi. MAITAMA has no bay post but a total of 25 staff at the call centre, known as the “compound unit”.
ii) *The ambulance services:* Ambulances used by FRSC were mainly ICU buses. NEMA has specialized ICU vehicles, 4 helicopters and 10 Boats for the riverine emergency bay post units. MAITAMA has less ambulance buses as most of them were grounded. All emergency victims and their relatives were conveyed to the final care facility by the organizations ICU ambulances. FRSC and NEMA- ICU ambulances were equipped with oxygen tanks and a few consumables (for example oxygen supply gear, gloves, shears, bandages, hand gloves, ECG monitoring machine, extraction machine, BCLS and ACLS equipment). Each ambulance bus takes up to 4 patients. MAITAMA ambulances lack extraction equipment. NEMA is the only EMRS organization with operational helicopters and boats, FRSC has helicopters but they are not functional, as these are grounded because of limited manpower and technical faults. FRSC and NEMA have 200 motorcycles operating 24 h a day and 7 days a week on the roads and suburbs of Abuja. NEMA has ambulance boats operating along the river that runs through and out of Abuja. (The Abuja River is a recreational spot, and it is also where the great water work of Abuja is located, supplying the city with water). The boats have basic emergency life support equipment. The hospital in MAITAMA runs seven ambulance buses. There are 21 additional buses, but they are grounded which limits the organizational effectiveness in response to emergencies.
iii) *Ambulance staff*: Each ambulance bus from FRSC and NEMA has a nurse and 3 paramedics when embarking on an emergency rescue. MAITAMA hospital has a complete team, made up of 1 doctor, 3 nurses and 2 paramedics. These are qualified staff in advanced cardiovascular life support (ACLS), which refers to a range of clinical interventions for the urgent treatment of cardiac arrest, stroke and other life- threatening medical emergencies; they also have the knowledge and skills to deploy advanced trauma life support (**ATLS**). However, there was a shortage of staff training: The total number of staff trained annually in emergency courses, workshops and seminars varies among the three organizations, as indicated in Table 2; 10% of the personnel from FRSC, 30% from NEMA and 8% of the staff from MAITAMA received such training during the last 12 months.
iv) *Cost of services*: Although the emergency services rendered are free of charge, the victims or relatives pay for consumables and blood. The cost is between150–400 Euro depending on the nature of the emergency and the organization involved in the rescue mission. This information was obtained from the heads of the three EMRS organizations.

**Table 2:** Resources of rescue services and capacity building (Information from heads of EMRS Organizations in Abuja)

Item	FRSC	NEMA	MAITAMA
Number of Call Centres	1	1	1
No of Ambulances & other vehicles.	8 ICU Buses	ICU 8, Special ICU Vehicles 8, Helicopters 4, Boats, 10	7 ICU functional ambulances (21 grounded)
No. of Bay post	13	10	0
No. of staff per Bay post	10	20	25(only at call Centre)
EMRS Basic equipment in ambulances	7/8	30/30	7/7
No. and % of trained staff/year	1 (10%)	6 (30%)	4 (8%)
Staff with Trained skills in ACLS, ATLS	5 (50%)	7 (35%)	4 (16).

ACLS: Advance cardiac life support machine

ATLS: Advance trauma life support machine

BLS: Basic life support machine

ICU: Intensive care unit

### Magnitude of medical emergencies and response by EMRS organizations

**Fig. 1:**
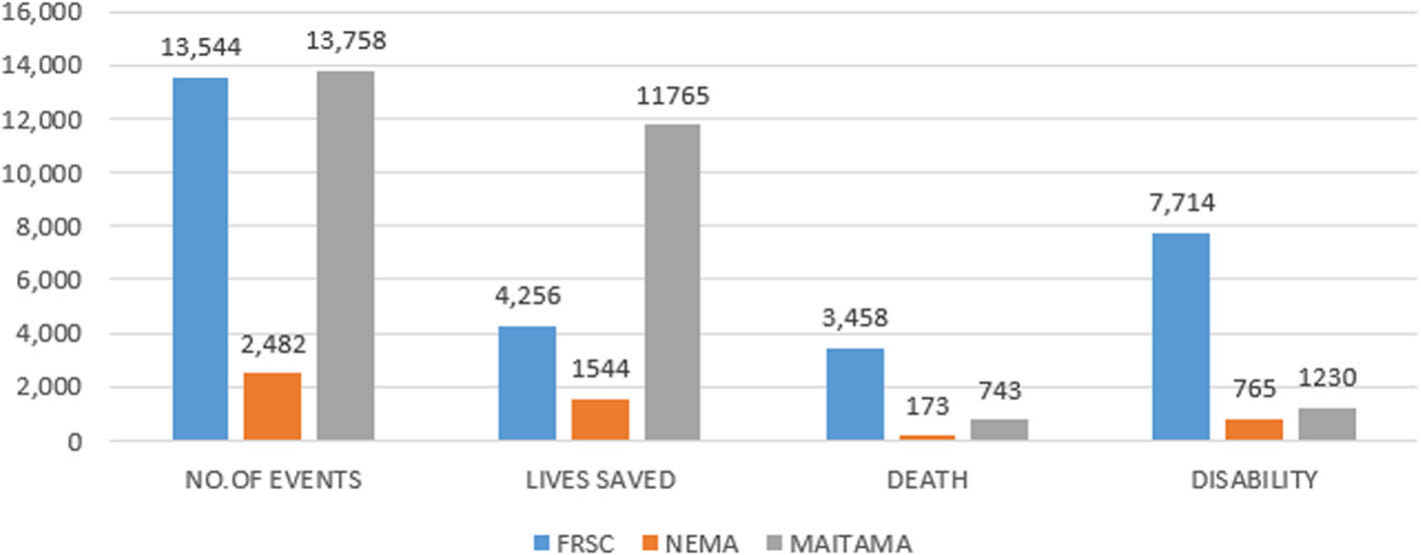
The magnitude of medical emergencies and rescue results (document analysis)

The documented amount of rescue operations carried out by FRSC and MAITAMA are shown in Fig. 1: approximately 13,600 to 13,700 operations in 2016; NEMA had a lower workload with less than 3000 operations (Fig. 1). The highest case fatality rate (CFR) occurred in traffic injuries (25.5% of all accidents), less in clinical emergencies (5.4%) and natural disasters (1.3%). The proportion of disabilities caused by traffic injury in FRSC (57%) was also higher than those by other medical emergencies (9% medical emergencies in MAITAMA and 30% in NEMA).

### The magnitude of medical emergencies and outcomes during the 14-days observation period

### The magnitude of medical emergencies and outcome during the 14-days observation period in each institution

Table 3 presents the activities observed by the 3 organizations regarding the number of calls, types of emergencies, number of lives saved, number of deaths and disabilities assessed during the 14-day observational study. It shows a high workload of 1000 calls a day in MAITAMA hospital mainly non - communicable diseases (stroke and other cardiovascular disease [CVDs], diabetic coma, snake bites) and a smaller number of road traffic injuries (RTIs). All calls at FRSC (192 per day) were related to RTIs and to a lesser extent to other medical emergencies. NEMA responded to an average of 87 NCD calls per day and to fewer RTI calls. The deaths in FRSC (3.6% fatality of all calls) were the lowest compared to higher fatalities in MAITAMA (4.1%) and NEMA (8%).

**Table 3:** EMRS and outcomes in two weeks observation period

INSTITUTION /ORGANIZATION	NO.OF CALLS PER DAY	TYPE OF EMERGENCY			TOTAL NO. OF EMRS	L IVES SAVED	DEATHS (%) (CFR)	DISABILITY (%)
		NCD	RTI	OTHER				
FRSC	80–192	2	190	–	192	125	7 (3.6%)	17 (8.9%)
NEMA	50–87	84	3	–	87	75	7 (8%)	5 (5.7%)
MAITAMA	500–1107	707	400	–	1107	1027	46 (4.1%)	110 (9.9%)
TOTAL	1386	793	593		1386	1227	60 (4.8%)	132 (10.8%)

*Case Fatality rate (CFR)

A total of 1386 emergencies occurred during the observation period, 1227(88.5%) lives were saved in the three organizations during the 14-day observation period, 60(4.9%) deaths were recorded, 132(9.6%) disabilities, 793(54.4%) were non- communicable diseases and 593(42.9%) were RTIs.

### Delay times of pre-clinical care (“transport delay”from call to arrival of ambulance at scene)

The speed of rescue services is crucial for the survival of the patient and the delay between accident/rescue-event and arrival of the rescue team or transport to specialized treatment in hospitals should be 15 min or less [1].

Table 4 shows the average delay times observed during the observation period of 14 days. The delay times from point of call to arrival at the scene (“transport delay”) for MAITAMA and NEMA were 15-30 min, FRSC delay times were much longer (over 35 min). FRSC is the agency responsible for RTIs, which often occur at the outskirts of Abuja. It is imperative (as explained by the heads of the various EMRS organizations) that emergency rescue victims from FRSC and NEMA are conveyed to MAITAMA hospital at most instances because it is a government institution, with a well-trained team.

**Table 4:** Response time (in minutes) from the event to rescue to clinical care in Abuja as measured during 14 days in each institution

INSTITUTION	AMBULANCE TYPE	EMERGENCY TYPE NO.& GENDER OF VICTIMS				DEATHS (CFR)	RESPONSE TIME (Average Mins). (± SD)
		TOTAL M F					
FRSC (VR)	ICU BUS	RTA	192	50	142	7 (3.6%)	35(±7)
NEMA (VR)	ICU/SP.BOAT	NCD/RTA	84	34	50	7 (8.3%)	16(±7)
MAITAMA (VR)	ICU BUS	NCD/RTA	1107	300	807	46 (4.2%)	15(±7)

*Case Fatality rate (CFR)

### Activities of rescue staff at scene of emergency

First aid activities carried out by EMRS staff of the 3 institutions at the scene include the administration of analgesic or pain killers, the arrest and stitching of wounds, the extraction of victims or dead bodies (mostly by FRSC and NEMA, but not by MAITAMA). Painkillers and other medicines are administered, an oxygen mask is placed on the face of the victim when the patient cannot breathe properly and the heart rate and volume are monitored using the ECG machine in the ambulance. MAITAMA has a clock which is placed in the ambulance to monitor time of arrival at scene and arrival time at the hospital for effective time management and disciplinary action in case of late arrival by the emergency team. The victim is finally transferred by means of a hospital stretcher or in some instances carried to the emergency unit, where care and management is given to the patient.

### Opinions of users on EMRS services and “call delays”

**Fig. 2:**
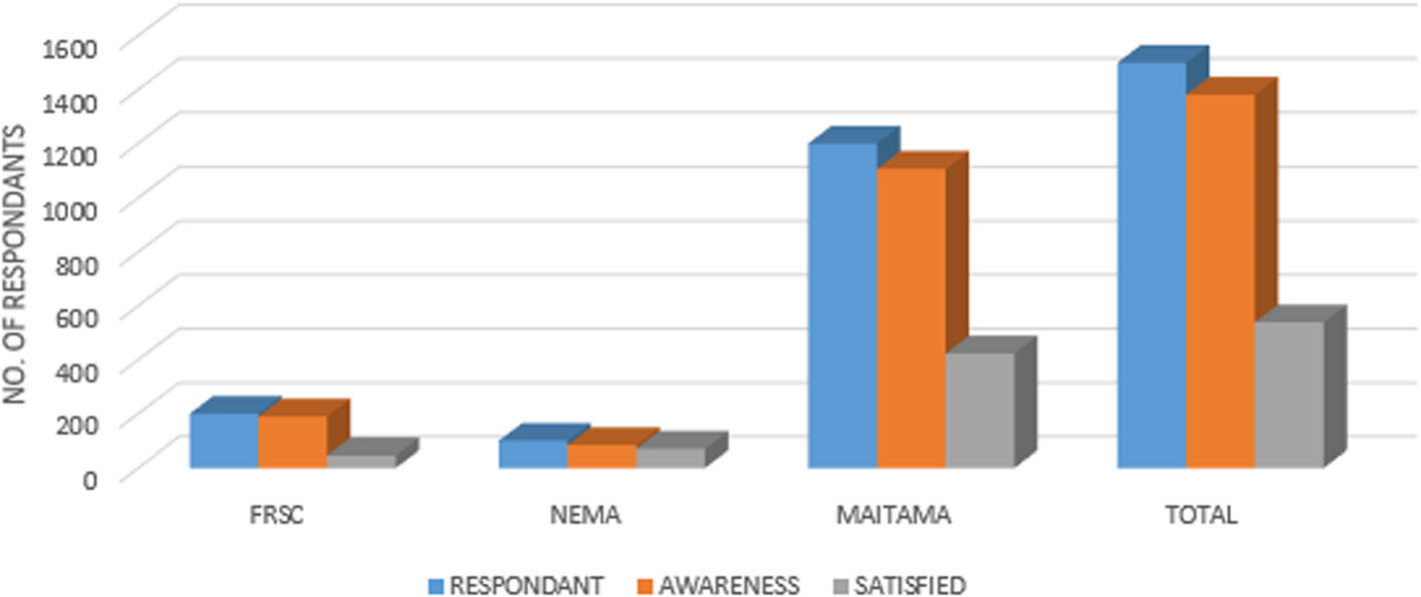
Opinions of users on EMRS services and “call delays”

Figure 2 shows that the majority of users knew about EMRS and emergency call numbers. Some of the victims, however, were unaware that such services existed, mainly victims coming to Abuja from other cities or states. Others mentioned in the interviews that they were concerned about potential treatment costs. One issue mentioned was that there was no unique call number and as a result it was difficult to identify the right number promptly, thus, the “call delays” (from start of the event until calling the emergency services). Furthermore, interference in telecommunication lines made it difficult to get through to the call centres.

Satisfaction regarding EMRS rendered by the three organizations were low for FRSC and MAITAMA, 23 and 36% respectively. However, satisfaction with NEMA services was quite high (70%). The delay times until arrival of the rescue team, insufficient supplies and medications were frequently mentioned as a cause of dissatisfaction. Victims who used MAITAMA hospital ambulances also mentioned the lack of health insurance for EMRS victims. FRSC and NEMA services are free, but users are charged for the treatment at MAITAMA hospital. Most users said that they prefer to use the services of the few private EMRS centers in Abuja in comparison with government services because of dissatisfaction with the latter, in particular the question of responsiveness and staff attitude. Very few victims or relatives came by private cars or taxis.

## Discussion

Pre-clinical care is an integral part of a national health care system [17]. In this study we quantified the burden of medical emergencies (morbidity and case fatality) in Abuja/Keffi, the existing EMRS using input, process, output and outcome indicators and identify options for improvement (Gap Analysis). This is the first study (public or private) to be carried out in Nigeria on emergency medical rescue services, considering the 6 key medical emergency stages [18–20].

### EMRS burden (document analysis and observation)

An enormous frequency (incidence) of emergencies was documented in 2016 with a case fatality of 3.5% for FRSC, 1.1% for NEMA and 3% for MAITAMA hospital, showing the highest figures for RTIs which is in line with other studies [16, 21]. However, a total of 1227 out of 1386 (88.5%) lives were saved by the 3 organization during the 14-days observation period in the 3 institutions.

### Emergency response (observation study)

During the observation study period using the 6 key stages of emergency, it was observed that nearly 90% of victims were brought within the first hour from the start of the emergency event to the hospital in spite of traffic congestion. This is still a long time for most victims but it is a substantial improvement compared to previous times. In the 1990s in Nigeria, private ambulances were mostly involved in EMRS services and 94% of all transports had long delays [8, 22, 23]. Currently, almost all emergency transport services are rendered by public ambulances often at reasonable speed.

In this study, we distinguished between “call delay” (from occurrence of the event until calling the call centre) and “transport delay” (from receiving the call at the centre to arriving at the hospital/health care facility). The call delays were obtained through observational periods and interviews with victims. It was noticed in the interviews that long call delays were due to possible treatment costs, lack of a unique emergency number and communication/network interferences which made it difficult to get through to the call centre. Many of victims interviewed were not sure which of the 3 EMRS organizations to call and their various roles and functions leading to extended call delays. The “transport delay” (from call to the rescue team arriving at scene with first aid interventions) as measured in this study was generally acceptable to be good (15 to 30 min for MAITAMA and NEMA; it was longer for RTIs as FRSC is frequently rescuing victims outside Abuja city). The delay times were shorter in NEMA and MAITAMA because of better ambulance services. Compared to other Low and Middle Income Countries (LMICs) like Ghana, Bangladesh and other developing countries, the delay ranged between 30 min to 72 h [1].

### Quality of infrastructure and equipment

The quality of EMRS of the 3 institutions in Abuja was satisfactory compared to other LMICs like Bangladesh (Dhaka) and Togo [16, 17, 24, 25]. These could be attributed to the establishment of FRSC, NEMA and MAITAMA in the late 1980s. FRSC and NEMA have a unique structure of emergency centres known as “Bay Posts”. They are located every 17 km along major roads in Abuja and other parts of Nigeria. Bay posts are well equipped with basic ambulances, trained staff, security and staff with motorcycles which pave the way in obstructed/congested roads for the ambulances during emergencies. The ambulance services are free of charge. However, Abuja suburb is highly populated and when emergencies occur in these areas, delays due to traffic congestion, bad road networks and poor communications contributed to late arrival at the hospital/health care facility.

### Staff skills

FRSC, NEMA and MAITAMA have trained staff comprising Paramedics, Nurses and Doctors. However, having a skilled team does not equate the existence of a high quality EMRS system. Regular on-the–job-training is needed. As elicited in the 3 EMRS agencies, only 20% of staff had such a training in the preceding 12 months. The attitude and behavioral aspects of the various EMRS staff of the 3 organization need to be addressed as many EMRS victims complained about unfriendly staff behaviour.

### Limitation of the study

One limitation of this study was that those who died during transport to the hospital or at the RTIs scene could not be analyzed separately. Secondly, the three EMRS organizations are not the only agencies in Abuja that offer emergency services. Other emergency medical rescue institutions, such as the Fire Services, the Red Cross, the Federal Civil Service Defense Corps and other private EMRS, need to be assessed and included in future studies.

## Conclusions

The outcome of acute illness or injury is strongly influenced by early recognition of its severity, fast activation of a call centre, rapid arrival of first aid and the fast transport to clinical care. Since most emergencies start at home, any system to promote the early recognition of emergency conditions should be based in the community. The study established that ambulances services are often out of reach for rural and suburb inhabitants. There is limited information on the ability of lay persons and community health workers to recognize and deal with life-threatening emergency conditions. However, it is reasonable to train individuals at suburbs and rural communities to recognize for instance severe blood loss in a postpartum woman, or breathing difficulty in an infant. Many of the benefits of pre-hospital emergency care could be realized by training community volunteers in simple but vital interventions, e.g. establishing and maintaining a patent airway, controlling external bleeding, and immobilizing fractures by means of local materials and resources [1, 25].

EMRS institutions need to have a unique call number. Such numbers should be promoted in the media and in the educational systems. Reducing call times and improving EMRS activities in Abuja will significantly reduce delay times and improve EMRS activities, thereby reducing deaths and disabilities.

## Data Availability

Data is not restricted, data are contained in the manuscript.

## Abbreviations

A&E: Accident and Emergency
ACLS: Advanced Cardiac Life
ATLS: Advanced Trauma Life Support
BLS: Basic Life Support
BSF: Bjorn Steiger Foundation
CFR: Case Fatality Rate
CVD: Cardiovascular Diseases.
DALYS: Disability-Adjusted Life-Years
EMRS: Emergency Medical and Rescue Services
FGD: Focus Group Discussion
FRSC: Federal Road Safety Commission
ICU: Intensive Care Unit
LMICs: Low and Middle Income Countries
NCD: Non-communicable Diseases.
NEMA: National Emergency Management Agency
RTI: Road Traffic Injury
WHO: World Health Organization
